# Bio-functional hydroxyapatite-coated 3D porous polyetherketoneketone scaffold for enhanced osteogenesis and osteointegration in orthopedic applications

**DOI:** 10.1093/rb/rbae023

**Published:** 2024-03-14

**Authors:** Huanhuan Liu, Taiqing Liu, Zhicheng Yin, Xiaoyin Liu, Ying Tan, Yuwei Zhao, Haiyang Yu

**Affiliations:** Department of Prosthodontics, State Key Laboratory of Oral Diseases, West China Hospital of Stomatology, Sichuan University, Chengdu, Sichuan 610065, PR China; Department of Pharmacy, State Key Laboratory of Biotherapy and Cancer Center, West China Hospital, Sichuan University, Chengdu, Sichuan 610065, PR China; Department of Prosthodontics, State Key Laboratory of Oral Diseases, West China Hospital of Stomatology, Sichuan University, Chengdu, Sichuan 610065, PR China; Department of Dental Technology, National Clinical Research Center for Oral Diseases, West China Hospital of Stomatology, Sichuan University, Chengdu, Sichuan 610065, PR China; Department of Neurosurgery, West China Hospital, West China Medical School, Sichuan University, Chengdu, Sichuan 610065, PR China; Department of Prosthodontics, State Key Laboratory of Oral Diseases, West China Hospital of Stomatology, Sichuan University, Chengdu, Sichuan 610065, PR China; Department of Prosthodontics, State Key Laboratory of Oral Diseases, West China Hospital of Stomatology, Sichuan University, Chengdu, Sichuan 610065, PR China; Department of Prosthodontics, State Key Laboratory of Oral Diseases, West China Hospital of Stomatology, Sichuan University, Chengdu, Sichuan 610065, PR China

**Keywords:** 3D printing, polyetherketoneketone, porous scaffold, hydroxyapatite, osteogenesis

## Abstract

Polyetherketoneketone (PEKK), a high-performance thermoplastic special engineering material, maintains bone-like mechanical properties and has received considerable attention in the biomedical field. The 3D printing technique enables the production of porous scaffolds with a honeycomb structure featuring precisely controlled pore size, porosity and interconnectivity, which holds significant potential for applications in tissue engineering. The ideal pore architecture of porous PEKK scaffolds has yet to be elucidated. Porous PEKK scaffolds with five pore sizes P200 (225 ± 9.8 μm), P400 (411 ± 22.1 μm), P600 (596 ± 23.4 μm), P800 (786 ± 24.2 μm) and P1000 (993 ± 26.0 μm) were produced by a 3D printer. Subsequently, the optimum pore size, the P600, for mechanical properties and osteogenesis was selected based on *in vitro* experiments. To improve the interfacial bioactivity of porous PEKK scaffolds, hydroxyapatite (HAp) crystals were generated via *in situ* biomimetic mineralization induced by the phase-transited lysozyme coating. Herein, a micro/nanostructured surface showing HAp crystals on PEKK scaffold was developed. *In vitro* and *in vivo* experiments confirmed that the porous PEKK-HAp scaffolds exhibited highly interconnected pores and functional surface structures that were favorable for biocompatibility and osteoinductivity, which boosted bone regeneration. Therefore, this work not only demonstrates that the pore structure of the P600 scaffold is suitable for PEKK orthopedic implants but also sheds light on a synergistic approach involving 3D printing and biomimetic mineralization, which has the potential to yield customized 3D PEKK-HAp scaffolds with enhanced osteoinductivity and osteogenesis, offering a promising strategy for bone tissue engineering.

## Graphical Abstract

**Figure:**
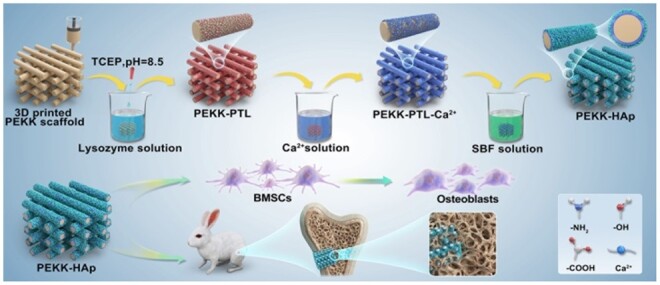

## Introduction

Polyetherketoneketone (PEKK) has been gradually used in the manufacture of orthopedic implants because of its excellent mechanical properties, radiolucency and chemical resistance. However, the hydrophobic surface and biological inertness of PEKK tend to limit osteointegration with the surrounding natural bone [1, 2]. The osseointegration potential of an implant is commonly understood to depend on its osteoconductivity and osteoinduction [3]. To address the above challenges, based on additive manufacturing techniques and surface modification methods, porous scaffolds with fully interconnected pores and biomimetic coatings were fabricated, which could provide appropriate biomechanical support for bone repair and, ultimately, coexist with the ingrowth of newly formed tissue [4–6]. 3D printing technology has garnered growing interest for producing controllable architectures by flexibly and accurately regulating the dimensional parameter of products [7, 8]. It has unleashed enormous potential in the fabrication of personalized scaffolds. Recently, it has been reported that porous scaffolds with profitable mechanical properties can be manufactured by 3D printing technology [9, 10]. Despite numerous relevant studies focusing on the cellular response and osteointegration of porous scaffolds with various pore sizes, a consensus on the optimal pore size for the mechanical and biological properties of scaffolds has yet to be reached [11–13]. Literature suggests that scaffolds with a pore size <100 μm may not adequately facilitate mass transport and cell migration, and a pore size >300 μm is favorable for bone formation via vascularization [14]. Therefore, for better osteointegration, within the recommended values, the pore size of the scaffold must be appropriate to ensure mechanical integrity and fulfill the needs for nutrient and waste diffusion of the tissue.

Although the porous scaffold enables more facile ingrowth of newly formed tissue, the limited bioactivity and inferior osseointegration with bone tissues due to the biological and chemical inertness of materials restrict its application in orthopedics. To facilitate enhanced osseointegration and promote robust bone formation, strategies have been devised to enhance the biocompatibility and bioactivity of PEKK [15–17]. Surface modification is an effective approach for improving biological properties without compromising mechanical strength and damaging advantageous bulk properties [18, 19]. Hydroxyapatite (HAp), as a constituent of natural bone, has been extensively employed in hard tissue engineering to achieve better bioactivity of implant surfaces [20–22]. HAp coatings can be developed by various deposition techniques, including plasma spraying [23], ion-beam-assisted deposition [24] and electrospray deposition methods [25]. However, all of these techniques have deficiencies in regard to HAp coating on scaffold surfaces. Specifically, the plasma spray method utilizes elevated temperatures ranging from 10 000 to 12 000°C, and the deposition process of HAP powder on the material surface is faster. The production conditions for ion-beam-assisted deposition and electrochemical deposition are rigorous and challenging to control. Consequently, the stringent and intricate production conditions present challenges in maintaining the quality, composition and crystallinity of the HAp coating. To avoid the limitations of these aforementioned coating methods, a biomimetic coating process stands out, it is a mild process using simulated body fluid (SBF) for biomimetic mineralization. The resulting coating of bone-like apatite on the porous scaffolds is dense and homogeneous [26]. Adequate surface modification is typically crucial to provide a firm bond form between the HAp coating and the substrate. Inspired by the natural phenomena of HAp formation and crystal organization facilitated by biomolecules, various methods of biomimetic mineralization for forming coatings on the surface of materials have been reported [27, 28]. The polydopamine (pDA) adhesion system has been used as a surface modification in numerous fundamental and applied research studies [18, 29]. However, the pDA coating is uneven and unstable, and the deposition process requires a relatively lengthy duration. Thus, modifying the surfaces via simple experimental procedures under mild reaction conditions is critical to facilitate HAp formation on complex porous PEKK scaffolds. Lysozyme undergoes a phase transition induced by tris(2-carboxyethyl) phosphine (TCEP), and subsequently transforms into an amyloid substance capable of adhering to the substrate surface, known as phase-transited lysozyme (PTL) [30]. The PTL membrane is enriched with carboxyl groups that enable chelation with calcium ions and could serve as a highly efficient template for facilitating the formation of a homogeneous HAp film. In contrast to conventional surface pretreatment methods, PTL-modification represents a novel approach for easily and inexpensively modifying complex-shaped materials.

**Scheme 1.**
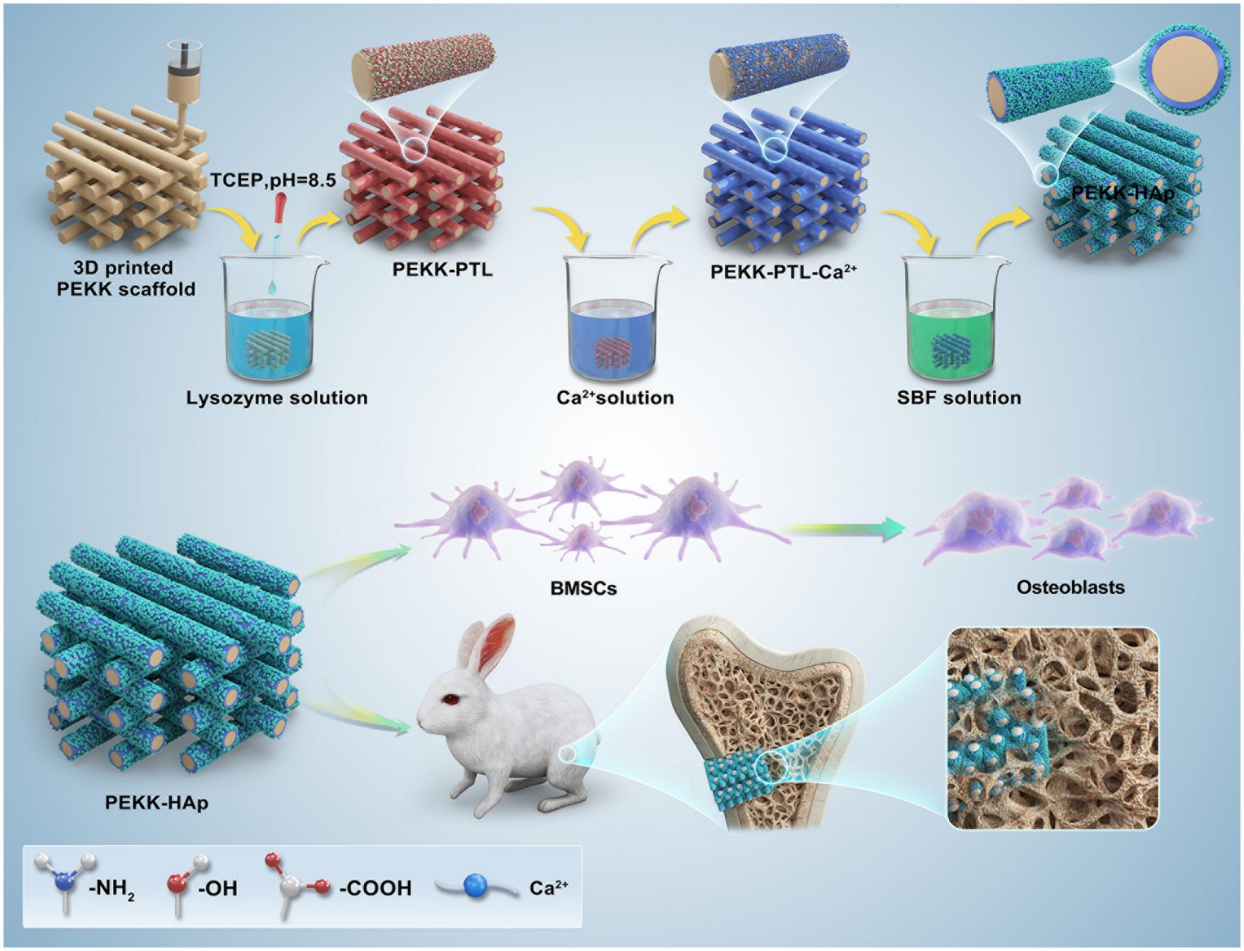
Schematic illustration of the preparation steps to produce the PEEK-HAp scaffold and its function in facilitating bone regeneration.

In this study, the advantages at the macro- and micro-levels were combined to synergistically promote the osseointegration of PEKK. As shown in Scheme 1, 3D printing technology was employed for the fabrication of an initial porous scaffold with optimal pore size. Subsequently, TCEP efficiently broke the disulfide bonds in lysozyme, and PTL coating was first employed to functionalize the surface of PEKK via all-purpose adhesion capability. Later, HAp crystals were bound to the PTL-coated PEKK scaffold surface using a biomimetic process, forming a functional micro/nanostructured surface. The modified PEKK (PEKK-HAp) scaffold was systematically investigated for its physical and chemical characteristics, *in vitro* cell response, feasibility and safety for bone repair in a femoral defect.

## Materials and methods

### Fabrication and characterization of 3D-printed PEKK scaffolds

These PEKK scaffolds were additively manufactured via a fused deposition modeling (FDM) 3D printer machine (Jugao-AM-Doctor, Shaanxi Jugao-AM Technology Co, Ltd, China) using PEKK filaments (Victrex, UK). The 3D models of PEKK scaffolds were designed using computer-aided design (CAD) software UG NX 10 (Siemens PLM Software, Germany). Supplementary Figure S1 showed the CAD-designed five experimental groups of porous PEKK scaffolds with flat shape, including pore sizes of 200, 400, 600, 800 and 1000 μm (denoted the P200, P400, P600, P800 and P1000, respectively). Then, the designed specimens were 3D printed using the FDM technique. The fabrication parameters, such as a 0.4-mm diameter nozzle, a printing speed of 40 mm/s, a printing line width of 0.4 mm, a layer thickness of 0.2 mm and an extrusion head nozzle temperature of 420°C, were fine-tuned through preliminary experiments for optimization.

The microstructure of the porous PEKK scaffold sections was characterized through environmental scanning electron microscopy (JSM-IT500, JEOL, Japan). The actual pore size was measured by ImageJ software based on the SEM images. The actual porosity of the scaffolds was calculated according to the ethanol immersion method based on Archimedes’ Principle as previously described [31]. Briefly, the porosity (*P*) was calculated using the equation: *P*=(V1 - V3)/(V2 - V3) × 100%, where V1 represented the initial volume of the graduated cylinder containing ethanol and the scaffold, V2 denoted the volume after ethanol penetration into the scaffold pores until no bubbles were observed under vacuum, and V3 indicated the remaining ethanol volume following scaffold removal. Thus, the scaffold’s porosity was expressed as the ratio of pore volume to the total volume of the scaffold, including pores, represented as a percentage. The mechanical strength was explored using a universal testing machine (Instron 3400, MA, USA) at ambient temperature. Uniaxial compression tests were performed on the scaffolds to evaluate compressive properties with a load of 250 N and a crosshead speed of 1.0 mm/min. And three-point bending strength of the scaffolds was assessed using a cross-head speed of 0.5 mm/min. Force–displacement data were collected to determine the ultimate stress and modulus, as well as to construct stress–strain curves.

### Cell proliferation and morphology

Bone marrow stromal cells (BMSCs) were seeded on the scaffolds in 24-well plates with complete medium (DMEM supplemented with 10% fetal bovine serum, 100 U/mL penicillin, and 100 μg/ml streptomycin) at a density of 2 × 10^4^ cells/well. Cell Counting Kit-8 (Dojindo, Japan) was used to evaluate cell proliferation. At 1, 4 and 7 days, a CCK-8 solution comprising 10% of the medium volume was added to each well and incubated for 1 h. Then, the absorbance value of the incubated solution was measured at 450 nm with a spectrophotometric microplate reader (Multiskan GO, Thermo, USA). After cells were seeded onto the scaffolds for 4 days, cell viability was assessed through live/dead cell staining using Calcein-AM/PI Double Stain Kits (Invitrogen, USA). Subsequently, fluorescence images were captured randomly using a confocal laser scanning microscope (CLSM, FV3000, Olympus, Japan).

After 1 and 4 days of incubation, the scaffolds were rinsed with PBS and then fixed in 2.5% v/v glutaraldehyde for 30 min. Subsequently, the scaffolds underwent dehydration via an ethanol series. Finally, cell morphologies on the scaffolds were observed using SEM.

### Alkaline phosphatase activity and extracellular matrix mineralization

Alkaline phosphatase (ALP) activity and extracellular matrix (ECM) mineralization were measured to determine the osteogenic differentiation of rBMSCs. Following a 48-h coincubation with the scaffolds, the culture medium was replaced with osteogenic inductive medium (complete medium with 10 mM β-glycerophosphate, 50 μg/ml ascorbic acid and 10 nM dexamethasone). ALP staining was conducted after culture for 7 and 14 days. The samples were washed with PBS, fixed with 4% paraformaldehyde, and subsequently stained for 30 min using a 5-bromo-4-chloro-3-indolyl-phosphate/nitro-blue tetrazolium (BCIP/NBT) Alkaline Phosphatase Color Development Kit (Beyotime Biotechnology, Shanghai, China). ALP activity in the cell lysate was determined by measuring the absorbance of the supernatant at 520 nm.

ECM mineralization was assessed by Alizarin Red S (ARS, Leagene Biotechnology, Beijing, China) staining for calcium nodules after 14 days. Cells were fixed with 4% paraformaldehyde and stained with a working solution for 30 min. In the quantitative assay, the samples were washed with distilled water to remove unbound dye. The bound stains were dissolved in 10% cetylpyridinium chloride (BOMEI Biotechnology, Hefei, China) in 10 mM sodium phosphate, and the absorbance values of the stained eluents were measured at 562 nm.

### Surface modification of porous PEKK scaffolds

Based on initial experimentation regarding mechanical and biological characteristics, a pore size of 596 ± 23.4 μm was selected as a constant for modification. The PEKK scaffolds were submerged in a solution containing a mixture of lysozyme solution (2 mg/ml lysozyme in 10 mM HEPES buffer) and TCEP solution at pH 8.5 (50 mM TCEP in 10 mM HEPES buffer), with a volume ratio of 1:1. After 2 h, a PTL coating was stably formed on the PEKK scaffold surfaces, denoted PEKK-PTL. The scaffolds were subsequently washed twice with distilled water to remove salts and additives adhering to the coating, followed by drying and vacuum storage. Subsequently, the PEKK-PTL samples were immersed in a 25-mM aqueous calcium chloride (CaCl_2_) solution for 24 h at ambient temperature. The obtained samples were named PEKK-PTL-Ca^2+^. After being gently washed with distilled water to remove Ca^2+^ without chelation, the samples were immersed in SBF at 37°C for 3 days. Throughout this duration, the SBF solution was replaced once every 24 h. In this process, HAp formed, followed by gently rinsing to remove the unimmobilized crystals and ions and drying under vacuum. Based on such PTL-templated interfacial biomineralization, the resulting HAp-decorated scaffolds called PEKK-HAp were obtained for further characterization.

### Characterization of modified scaffolds

The surface morphology and chemical composition of the scaffold surface of PEKK, PEKK-PTL, PEKK-PTL-Ca^2+^ and PEKK-HAp were examined by SEM with an X-ray energy dispersive spectrometer detector (EDS, JSM-IT500, JEOL, Japan). X-ray photoelectron spectroscopy (XPS, AXIS Supra, Kratos, USA) was utilized to identify the surface composition evolution of the scaffolds. The surface chemical structure of the samples was characterized by Fourier transform infrared spectrometry (FTIR, Nicolet 6700, Nicolet, USA). The crystallographic characteristics of the HAp coating on the scaffolds were determined using X-ray diffraction (XRD, D8 Advance, Bruker AXS, Germany). The 3D surface topography and roughness of the samples were analyzed using atomic force microscopy (AFM, SPM-9600, SHIMADZU, Japan) on the microscale in noncontact mode. Water contact angle (WCA) measurements were performed to characterize the surface wettability and hydrophilicity, determined by a contact angle system (Drop Shape Analyzer-DSA100, KRÜSS, Hamburg, Germany). Briefly, the shape of the water droplet was documented following the application of a 10-μl droplet onto the sample surfaces, and the contact angle was quantified.

### Preparation of extract from scaffolds

The extract solution from PEKK, PEKK-PTL and PEKK-HAp scaffolds was prepared according to the established protocol. Briefly, the scaffolds soaked in 10 ml DMEM were incubated in a humidified thermostatic cell incubator for 24 h. The aseptic supernatants were collected under sterile conditions, subjected to centrifugation at 5000 rpm for 5 min, and then filtered through 0.22 μm filter membranes before being stored at 4°C. Subsequently, the extracts were mixed with the corresponding medium at a volume ratio of 1:3 for cell culture.

### Biocompatibility of modified scaffolds

Cell adhesion, cell viability and cell proliferation were assessed following established methods. Specifically, rBMSCs were seeded onto the samples (PEKK, PEKK-PTL and PEKK-HAp) at a density of 2 × 10^4^ cells/well in 24-well plates. CCK-8 was used to evaluate cell proliferation at 1, 4 and 7 days. After 24 and 48 h, the morphology of the cells was characterized by SEM, as described in the section ‘Cell proliferation and morphology’. For fluorescein staining, F-actin was utilized to evaluate the cell morphology as described previously. In brief, cell culture media were replaced with different extractions obtained from the studied scaffolds and then incubated for 1, 4 and 7 days. Next, the cells were washed three times with PBS, fixed with 4% (w/v) paraformaldehyde for 15 min, permeabilized with 0.1% Triton X-100 for 30 min, stained with FITC-labeled phalloidin (Solarbio, Beijing, China) for 30 min, counterstained with 4′,6-diamidino-2-phenylindole (DAPI) and observed using CLSM.

### Scratch wound healing and transwell assays of rBMSCs

A scratch wound healing assay was conducted to evaluate the effects of extract from scaffolds on rBMSC migration activities. Briefly, rBMSCs were seeded at a density of 1 × 10^5^ cells/well in the 6-well plates and cultured for 2 days to reach confluence, subsequently, a straight line was made through the middle of every well by a sterile 200-μl pipet tip, and all cells were cultivated with the extract solution. After 0, 24 and 48 h, cells in each well were imaged using an inverted microscope (IX73, Olympus, Japan). The area of wound closure relative to the original area was quantitatively compared using ImageJ software.

Transwell assays were performed to further investigate rBMSC migration stimulated by the scaffolds. Different scaffolds (PEKK, PEKK-PTL and PEKK-HAp) were immersed in 800 μl of medium and then added to the lower chambers in the 24-well culture plates. A volume of 200 μl of rBMSC suspension containing cells at a density of 2 × 10^4^ cells/well in serum-free medium was added to the upper chamber. After 12 and 24 h of cultivation, cotton swabs were utilized to gently remove the non-migrated cells from the upper chamber. The cells that had migrated through the pores to the lower surface were then fixed using 4% paraformaldehyde for 15 min, and then subjected to staining with 1% (w/v) crystal violet. Subsequently, the migrated cells on the lower chamber were imaged using an inverted microscope. Finally, 400 μl of 10% acetic acid was employed to dissolve the crystal violet, and the absorbance of the supernatant was measured at 590 nm.

### Osteogenic activity of rBMSCs

rBMSCs were cocultured with different extracts and scaffolds at a density of 2 × 10^4^ cells/well, respectively. ALP activity and ECM mineralization were evaluated after osteogenic induction. These assays were performed per the protocols mentioned in the section ‘Alkaline phosphatase activity and extracellular matrix mineralization’. The expression levels of the osteogenesis differentiation-related genes ALP, collagen type 1 (COL1), osteocalcin (OCN) and runt-related transcription Factor 2 (RUNX2) were evaluated using quantitative real-time PCR (RT–qPCR). After a 14-day incubation period in osteogenic inductive medium, total RNA was extracted from rBMSCs using TRIzol reagent (Life Technologies, USA). Subsequently, cDNA was synthesized from RNA using a SensiFAST™ cDNA Synthesis Kit (Bioline, Australia) according to the manufacturer’s guidelines. PCR amplification was performed on an ABI 7500 machine (Applied Biosystems, CA, USA) using the SYBR premix EX Taq PCR kit (Takara, Japan) with specific primer sequences detailed in Supplementary Table S1.

### Immunofluorescence staining

Immunofluorescence was performed at 7 and 14 days after osteogenic induction to identify ALP and OCN proteins. After fixation and permeabilization, rBMSCs were preincubated with 1% bovine serum albumin for 30 min to mitigate nonspecific binding events. Subsequently, the samples were incubated with primary antibodies overnight at 4°C. Following PBS washes, the corresponding Alexa Fluor 594–conjugate anti-mouse secondary antibodies were applied to bind with the primary antibodies for 1 h. After PBS rinses, the cells were stained with FITC-labeled phalloidin for 30 min and then incubated with DAPI for 5 min for nuclear staining. Immunofluorescence images were captured using CLSM.

### *In vivo* repair of rabbit femoral defects

All animal experimental procedures were approved by the medical and experimental animal ethical review committee of Sichuan University (No. WCHSIRB-D-2023-399) and were conducted in strict adherence to the Committee’s guidelines. Male New Zealand white rabbits (12 weeks old) were used to establish a bone defect rabbit model. All surgical interventions were carried out under general anesthesia by phenobarbital sodium (3.5% w/v, 1 ml/kg) via ear vein injection. Briefly, 6 randomly selected rabbits were divided into three groups (PEKK, PEKK-PTL and PEKK-HAp), the surgical areas were shaved and sterilized, the muscles and fascia were separated through a skin incision of ∼2 cm, and the anterior-distal aspects of the femurs were surgically exposed. Defects measuring 5 mm in diameter and depth were created through drilling on the surfaces of the bilateral femurs. Different scaffolds were inserted into the cavity of the femur and then sutured layer by layer with a silk 3–0 suture.

### Micro-CT analysis

Eight weeks postoperatively, the femurs housing the implanted materials were subsequently retrieved for subsequent analysis. The samples were subjected to micro-CT analysis using a μCT device (vivaCT80, SCANCO Medical AG, Switzerland) to evaluate *in vivo* new bone formation. After scanning, 3D models were reconstructed and analyzed with SCANCO Medical Evaluation and Visualizer software. All reconstruction parameters were employed to assess new bone formation, predominantly encompassing bone volume relative to total sample volume (BV/TV), trabecular number (Tb. N), trabecular separation (Tb. Sp), and trabecular thickness (Tb. Th).

### Histological analysis

Following μCT analysis, the femoral samples underwent dehydration in a series of graded ethanol solutions, embedded in polymethylmethacrylate, and sliced ∼50 μm in thickness using a microtome (Leica Microtome, Germany). The sections were subjected to van Gieson (VG) staining and toluidine blue (TB) staining to detect the tissue response and bone ingrowth to the implanted materials. After sealing with neutral gum, observations and photography were performed using a Zeiss microscope (Carl Zeiss, Germany).

### Statistical analysis

All the data collected are presented as the mean±SD. Statistical software SPSS 20.0 was used to analyze the differences among groups by one-way analysis of variance followed by the Student–Newman–Keuls test. *P < 0.05, **P < 0.01 and ***P < 0.001 were defined as significant for all statistical tests.

## Results

### Structural characterization of porous PEKK scaffolds

**Figure 1.**
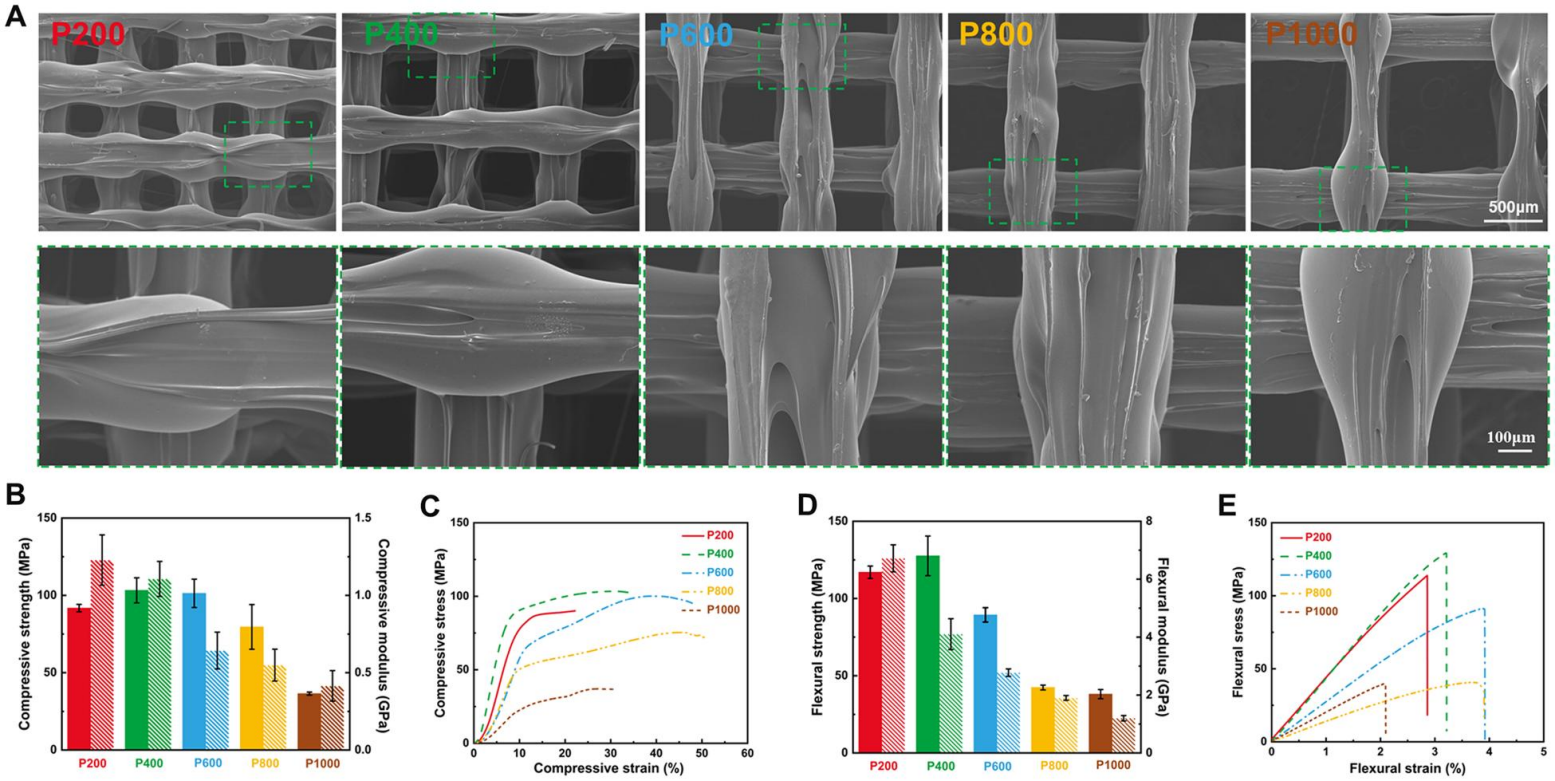
SEM images and mechanical characteristics of the 3D PEKK scaffolds. (**A**) SEM images of different scaffolds at different magnifications. (**B**–**E**) Mechanical properties of different scaffolds. (B) Compressive strength and modulus. (C) Compressive stress–strain curves. (D) Flexural strength and modulus. (E) Flexural stress–strain curves. Data are presented as the mean±SD (*n* = 3).

Porous PEKK scaffolds with regular and controllable architectures could be built up via the utilization of FDM. During the FDM, the PEKK product was melted by a high melting temperature to form a homogeneous dense filament, and the abovementioned parameters were input, leading to the additive manufacturing of porous scaffolds. Supplementary Figure S2 showed a general sketch of the samples. The SEM images revealed that the scaffold had a homogeneous 3D porous structure, and the pores exhibited a consistent quadrilateral morphology (Figure 1A). The struts were meticulously oriented, and the surface was sleek with sporadic impurities, which could be eliminated through ultrasonic cleaning. The mean pore diameter of the scaffold was ascertained using SEM, and scaffold porosity was quantified via ethanol immersion (Supplementary Table S2). Regarding scaffolds with uniform strut size, an augmentation in pore size from ∼200–1000 μm resulted in an increase in measured porosity from ∼24.6–58.6%. There was a difference of ∼40 μm between the pores of the as-produced scaffolds and the designed scaffolds. With regard to the limitation of printing accuracy, the error of pore size was inevitable but in the acceptable range.

The mechanical characteristics of the 3D PEKK scaffolds were assessed through uniaxial compressive strength analysis (Figure 1B and C) and three-point bending tests (Figure 1D and E). The results revealed a reasonable pore size dependence of the strength and the modulus, wherein the mechanical properties exhibited a consistent decrease as the pore size increased. Although there was a slight decrease in the strength of P200 compared to P400 due to localized minor collapse during printing, caused by limitations in printing accuracy. Typically, the compressive modulus and compressive strength, with pores at 200–800 μm, both fell within the range of yield strength and modulus of human trabecular bone from distal femur (Young’s modulus: 0.02–30 GPa, yield stress: 80–150 MPa). Flexural strengths also exhibited similar trends with respect to pore size for these samples. Referring to the 50–120 MPa flexural strength of human cortical bone, three strength values of the porous PEKK scaffolds fell within or beyond the above range, P200 (117.0 ± 4.0 MPa), P400 (127.6 ± 12.8 MPa) and P600 (89.4 ± 4.6 MPa). Accordingly, it was feasible to modulate the mechanical properties to a desired extent by fine-tuning the scaffold pore size. Meanwhile, the study also substantiated that optimizing the mechanical characteristics of porous biomaterials for bone implant applications to align with those of natural bone could reduce bone resorption caused by stress shielding. It was obvious that the porous scaffolds with 200–600 μm exhibited more appropriate strength and modulus for the orthopedic applications.

### Effects of scaffolds on cytocompatibility of rBMSCs and screening of the pore size of scaffolds

**Figure 2.**
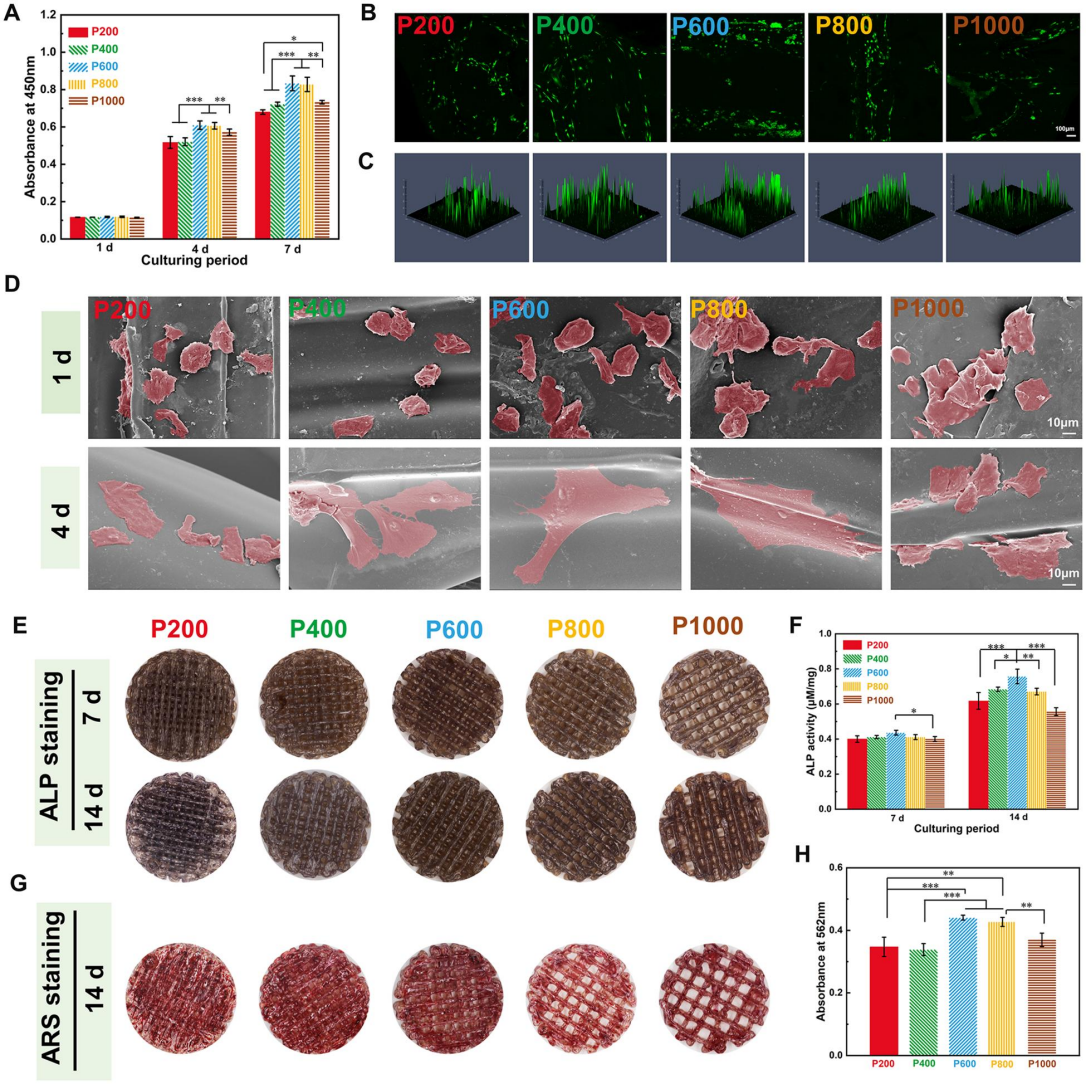
Proliferation, adhesion, differentiation, and mineralization of rBMSCs cocultured with different scaffolds. (**A**) CCK-8 of rBMSCs cultured for 1, 4, and 7 days. (**B**) AM/PI staining images of rBMSCs after 4 days of incubation. (**C**) Semiquantitative analysis of fluorescence intensity with ZEN 2.3 lite software. (**D**) SEM images showing the initial adhesion and spreading of rBMSCs incubated on the scaffolds for 1 and 4 days. (**E**) ALP staining of rBMSCs cultured for 7 and 14 days. (**F**) Quantitative ALP activity determination of cells. (**G**) ARS staining of rBMSCs cultured for 14 days. (**H**) Quantitative analysis of mineralized nodules (*P < 0.05, **P < 0.01 and ***P < 0.001).

CCK-8 assays were conducted to evaluate cell proliferation (Figure 2A). At 1 day, there were no significant differences in the cell viability among all porous scaffolds. After 4 days of incubation, cell proliferation was markedly increased in the five groups. Particularly, cell proliferation was notably higher in the P600 and P800 groups, with no statistically significant distinction between these two groups, followed by P1000, and P200 and P400 generated the lowest cell viability.

The fluorescence images of live/dead staining of rBMSCs cultured on the porous scaffolds were illustrated in Figure 2B. Live cells were represented by green fluorescence, predominantly presented on the strut surfaces, while red fluorescence, indicative of dead cells, was scarcely discernible. Consequently, these results indicated that porous PEKK scaffolds exhibited minimal cytotoxicity toward rBMSCs. Quantitative analysis using ZEN 2.3 lite software revealed that cells on P600 scaffolds displayed higher fluorescence intensity compared to other groups, indicating that PEKK scaffolds with a pore size of ∼600 μm were more favorable to cell proliferation (Figure 2C).

From SEM images in Figure 2D, healthy cells were observed to be distributed across the scaffold surfaces at 1 day. After 4 days incubation, cells exhibiting a well-dispersed pattern and polygonal morphology were observed on all scaffolds. Cells from the P600 and P800 groups displayed more lamellipodia extensions than the other groups, and the antennae of rBMSCs in the P600 and P800 groups were distributed and extended more widely than those in the other groups, indicating that the preferential adsorption of cells was influenced by pore size.

The effects of different porous PEKK scaffolds on the osteogenic differentiation of rBMSCs were evaluated through ALP activity and ARS staining. During the initial 7 days of culture, no significant differences were observed among the other porous scaffolds. Afterwards, the intensity of ALP staining increased uniformly across all scaffolds after 14 days of incubation. Notably, the ALP staining on the surface of the P600 group exhibited noticeably deeper compared to the other groups (Figure 2E). The corresponding quantitative analysis also confirmed that P600 generated the highest ALP activity, while the differences in ALP staining between the other groups were not significant (Figure 2F). Additionally, distinct patterns of calcium nodular deposits were discerned on the scaffold substrate in the P600 group at 14 days (Figure 2G). The corresponding quantitative analysis revealed that P600 generated the highest calcium deposition (Figure 2H).

These results indicated that the scaffolds had lower cell viability when the pore size exceeded 600 μm, and the larger the pore size of the scaffolds was, the lower the cell proliferation and osteogenic differentiation. Meanwhile, we found that rBMSCs were well attached on P600 and P800 scaffolds with numerous lamellipodia. The mechanical characterization parameters of the scaffolds exhibited a predominantly decreasing trend with increasing pore size. To ensure the high strength and low modulus of the scaffold as a support material, we would further study the P600 scaffolds to verify the changes in osteogenesis after modification.

### Surface characterization of modified scaffolds

**Figure 3.**
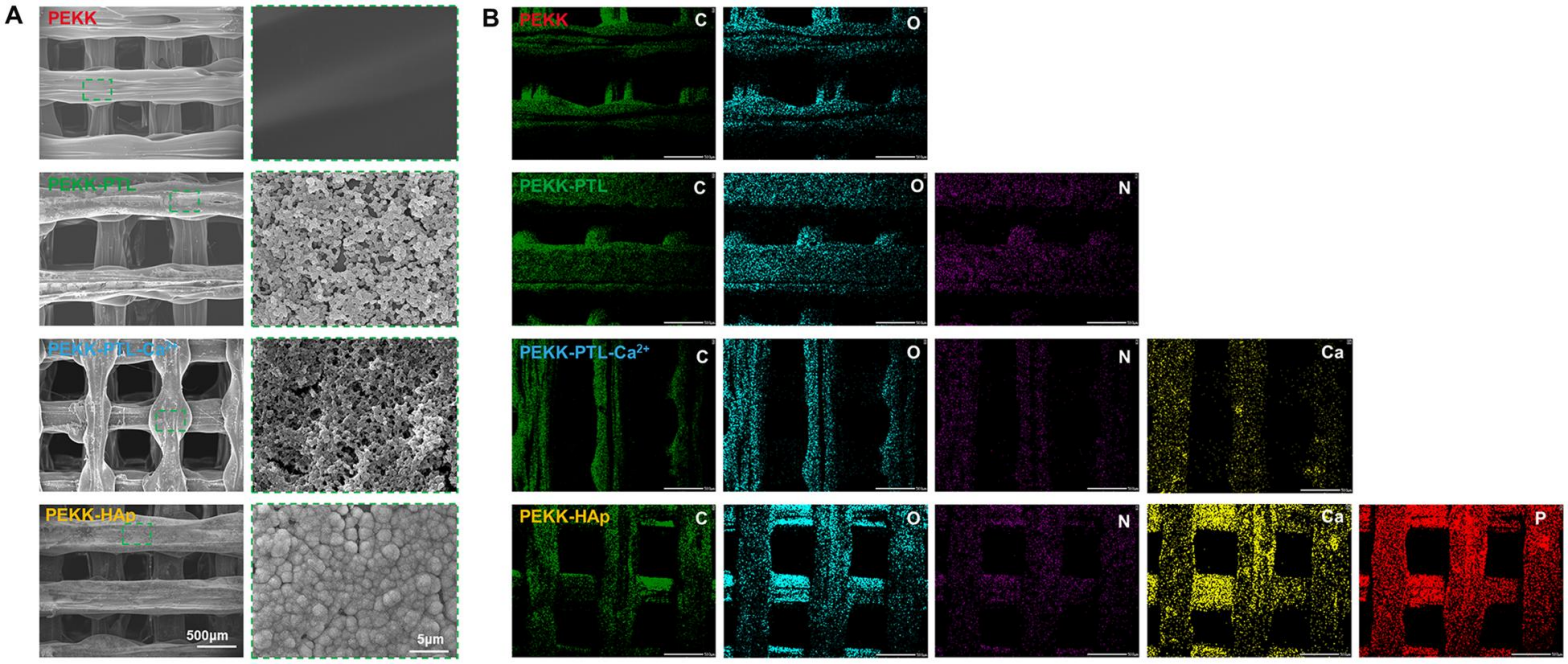
The formation of HAp induced by the PTL nanofilm. (**A**) SEM images of porous scaffolds at different magnifications. (**B**) Element mapping of different scaffolds, including C, O, N, Ca and P detected by EDS.

The PEKK scaffolds with HAp coating were fabricated as follows: Initially, the PTL coating was preferentially attached onto the PEKK surface, yielding a homogenous nanofilm and consequently generating a profusion of surface free radicals. Then, the coated substrate underwent immersion in a CaCl_2_ solution, resulting in the implantation of calcium ions onto the PTL surface. This was followed by transfer into SBF to facilitate the formation of a HAp layer and activate the surface. High-magnification SEM images revealed that the surface of pristine PEKK exhibited a predominantly flat and smooth topography. PEKK-PTL exhibited a homogeneous nanofilm surface morphology decorated with interconnected nanospheres. Following a 3-day incubation period in SBF, the scaffold treated with the PTL coating was fully covered by a homogeneous agglomerate layer featuring lath-like clustered protrusions, which are characteristic morphologies of HAp crystals (Figure 3A). EDS was then employed to analyze the elemental composition (Figure 3B). The results showed that C and O elements were the mainly chemical components of the untreated PEKK scaffold. For PEKK-PTL, N signal could be observed, corresponding to the hydrophobic amino acid residues from the PTL. Following the pretreatment with CaCl_2_ solution, uniform distributions of Ca were detected on the surface of the samples, indicating the successful chelation of Ca ions at the PEKK-PTL interface. Additionally, homogeneous distributions of Ca and P on the PEKK-HAp surface signified the effective coating of HAp.

**Figure 4.**
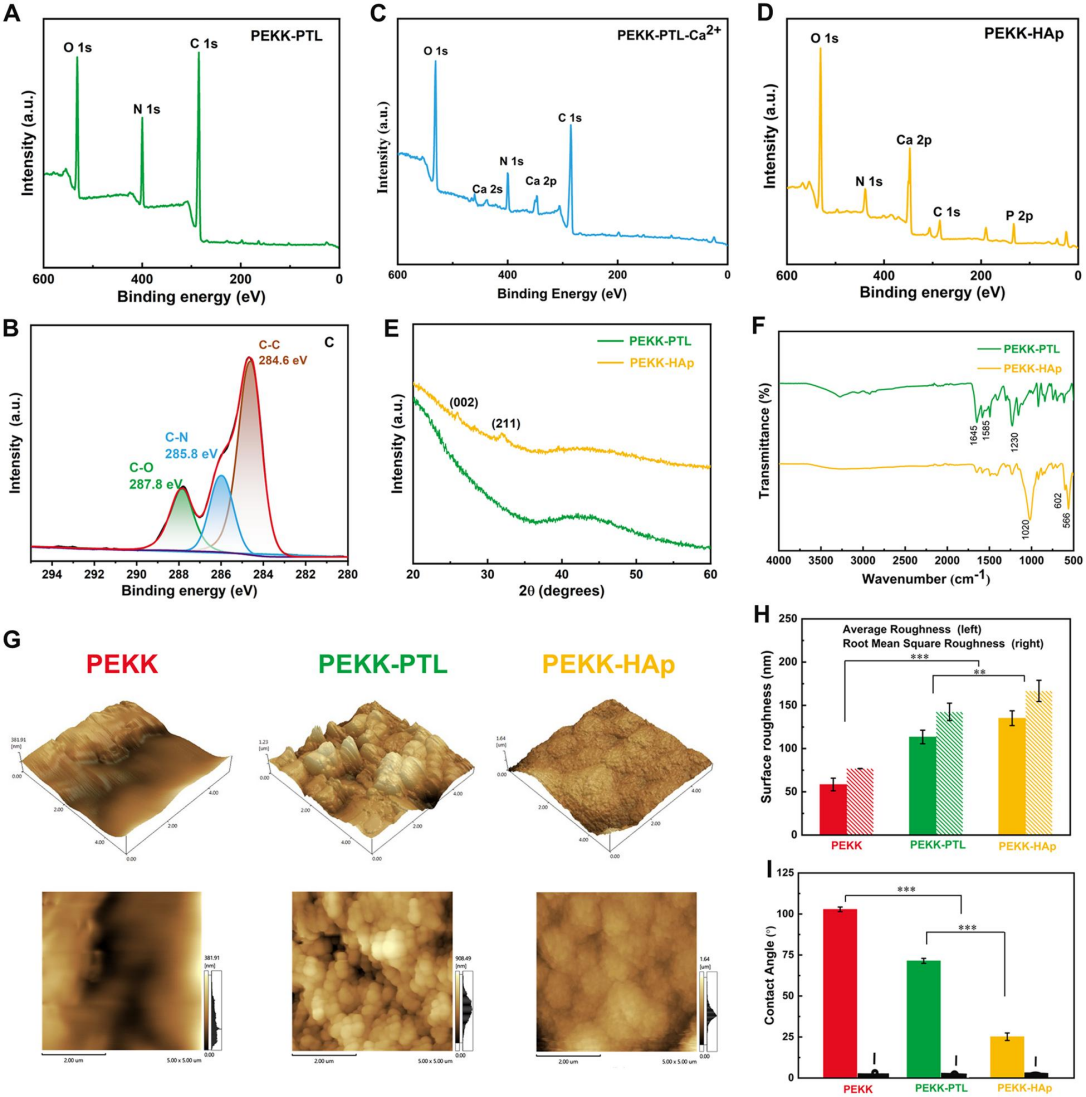
Surface morphology and structural analysis of different scaffolds. (**A**) XPS analysis of the PEKK-PTL scaffold. (**B**) High-resolution spectrum of C 1 s on PEKK-PTL. XPS analysis of (**C**) PEKK-PTL-Ca^2+^ scaffold, and (**D**) PEKK-HAp scaffold. (**E**) X-ray diffraction pattern of PEKK and PEKK-HAp scaffolds. (**F**) FTIR spectra of different scaffolds. (**G**) AFM images of different scaffolds. (**H**) Corresponding surface roughness analysis. (**I**) Water contact angle of different scaffolds (^*^P < 0.05, ^**^P < 0.01 and ^***^P < 0.001).

The chemical compositions of the biomaterial interface on the scaffold surfaces were further analyzed by XPS. The high-resolution C 1 s XPS spectra indicated that the PTL coating was effectively immobilized on the PEKK-PTL surface (Figure 4B). The high-resolution C 1 s spectra exhibited three primary peaks with binding energies of ∼287.8, 285.8 and 284.6 eV, attributed to C=O, C–O and C–C, respectively. The data indicated that the PTL coating surface exhibited an enrichment of carboxyl, amine and hydroxyl functional groups. Compared to the XPS spectra in the PEKK-PTL group (Figure 4A), a significant peak representing calcium (Ca 2p) emerged in the spectrum of PEKK-PTL-Ca^2+^ (Figure 4C) and PEKK-HAp (Figure 4D), demonstrating that PTL provided an active surface capable of chelating with Ca^2+^ ions through electrostatic interactions. Additionally, the high-resolution XPS spectra of Ca 2p and P 2p showed two predominant peaks in the Ca 2p spectrum (at 347.1 and 351.2 eV), corresponding to Ca groups, and a singular peak in the P 2p -spectrum (at 132.9 eV), corresponding to P groups within crystallin HAp (Supplementary Figure S3).

Furthermore, the XRD patterns of the resulting samples were illustrated in Figure 4E, providing confirmation that the two crystalline peaks corresponded to the characteristic diffraction peaks of HAp crystals, consistent with the diffraction patterns found in standard PDF cards. For the PEKK-HAp, a broad peak at ≈20° was the characteristic peak of amorphous PEKK, while the characteristic peaks of HAp [(201) and (211)] appeared at 25.9° and 31.8°. The data demonstrated that no new crystalline phase formed during the composite preparation process except for the inherent crystal structure of both PEKK and HAp.

FTIR spectra were used to determine alterations in chemical groups and characteristic bands of the different scaffolds, as shown in Figure 4F. Before the formation of HAp, the spectral signatures of PEKK-PTL exhibited peaks corresponding to amide I (C = O stretching) at 1645 cm^−1^, amide II (C–N stretching and N–H deformation) at 1585 cm^−1^, and amide III (C–N stretching and N–H deformation) at 1230 cm^−1^. After biomimetic mineralization in SBF, a marked attenuation of amide bonds was observed, indicating a noteworthy interaction between Ca^2+^ ions and the carboxyl groups of PTL. Meanwhile, the appearance of distinct peak positions in PEKK-HAp, specifically linked to O–P–O vibrations at 566, 602 and 1020 cm^−1^, suggested the formation of HAp crystals on the surface of the PEKK scaffold.

AFM image analysis was employed to conduct a more in-depth examination of surface morphology and roughness (Figure 4G). The results revealed that the initial application of the PTL coating significantly augmented the surface roughness of the scaffolds. Furthermore, the subsequent application of the HAp coating led to the appearance of more prominent surface protrusions, thus further amplifying the surface roughness (Figure 4H). The hydrophilic and hydrophobic evolution of the studied materials was characterized by WCA measurements. As depicted in Figure 4I, the PEKK surface exhibited poor wettability with a higher contact angle (102.8 ± 1.4°), whereas the contact angle of PEKK coated by PTL decreased to 71.4 ± 1.5° because of the hydrophilic recovery of polymers treated by phase transition. However, the contact angle on the PEKK-HAp surface decreased to 25.2 ± 2.3°, indicating that the HAp coating played a crucial role in increasing the surface hydrophobicity. This phenomenon could be attributed to the micro/nanostructure coupled with hydroxyl (OH) groups on the scaffold surface.

### Cell viability and morphologies

**Figure 5.**
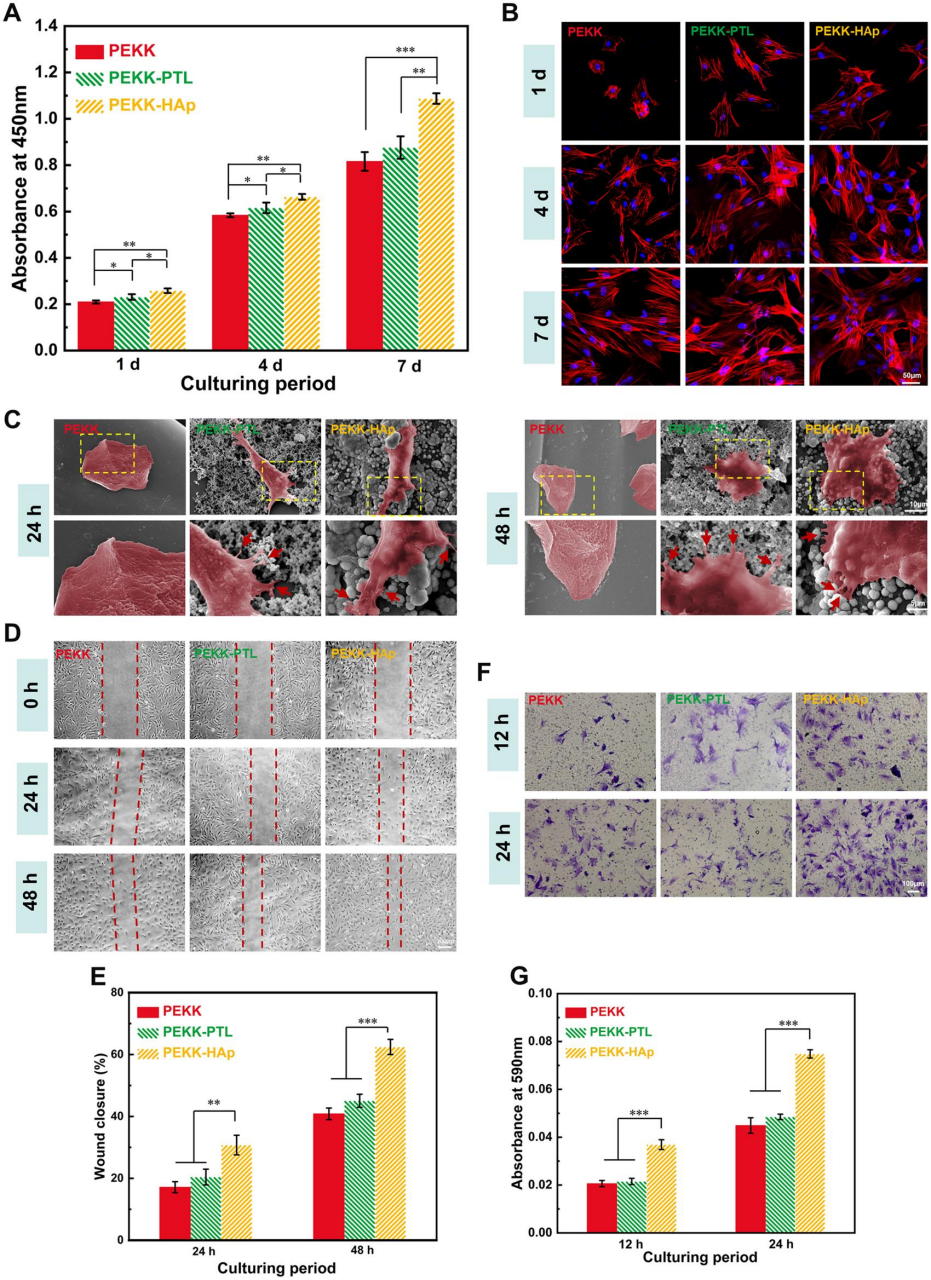
Proliferation, adhesion, and migration of rBMSCs cultured with different scaffolds. (**A**) CCK-8 of rBMSCs cultured for 1, 4 and 7 days. (**B**) CLSM images of rBMSCs cultured for 1, 4 and 7 days. (**C**) SEM images showing the adhesion of rBMSCs cultured for 24 and 48 h. Red arrows refer to pseudopodia of the cells. (**D**) Representative migration images of rBMSCs at different time points (0, 24 and 48 h). (**E**) Percentage of wound closure of different groups. (**F**) Transwell images of rBMSCs cultured with different scaffolds. (**G**) Quantitative analysis of migrated rBMSCs determined by crystal violet staining (^*^P < 0.05, ^**^P < 0.01 and ^***^P < 0.001).

Figure 5A illustrated a substantial increase in cell proliferation within the PEKK-HAp group relative to the other groups, indicating that the modified scaffolds facilitated enhanced cell proliferation. The cellular morphology, as observed via immunofluorescence, revealed cell adhesion and cytoskeletal arrangement in the different groups (Figure 5B). After 1, 4 and 7 days of culture, the different groups demonstrated a progressive increase in cell population and an enhanced degree of spreading, characterized by elongated and net-like lamellipodia. Specifically, this pattern was observed in the following sequence: PEKK < PEKK-PTL < PEKK-HAp. The images of SEM (Figure 5C) showed that noticeable cell clustering was observed on the PEKK substrate after culture for 24 h, indicating inadequate cellular adhesion to the unmodified surface. Conversely, the PEKK-PTL and PEKK-HAp groups demonstrated a spreading pattern of cells. Moreover, after culture for 48 h, more evident filopodia and lamellipodia were observed on PEKK-HAp than on PEKK-PTL. Hence, these results indicated that the biomimetic mineralization process used to coat the PEKK surface with HAp promotes favorable cell adhesion, thus providing superior conditions for subsequent cell proliferation.

### Cell migration

Figure 5D presented the outcomes of the scratch assay conducted under indirect contact coculture conditions between rBMSCs and different porous scaffold groups. Statistical analysis indicated that the wound healing effect in the PEKK-HAp group was significantly enhanced compared to that in the other two groups at 24 and 48 h, while no significant difference in cell migration healing effect was observed between the PEKK and PEKK-PTL groups (Figure 5E).

In addition, a transwell invasion chamber assay conducted within 24 h indicated that the migratory capability of rBMSCs in the PEKK-HAp group exhibited superiority over the remaining groups (Figure 5F and G). Collectively, these phenomena may be related to the fact that the presence of HAp coating enhanced cell adhesion, potentially contributing to the augmented motility and migration abilities [21].

### Osteogenic differentiation

**Figure 6.**
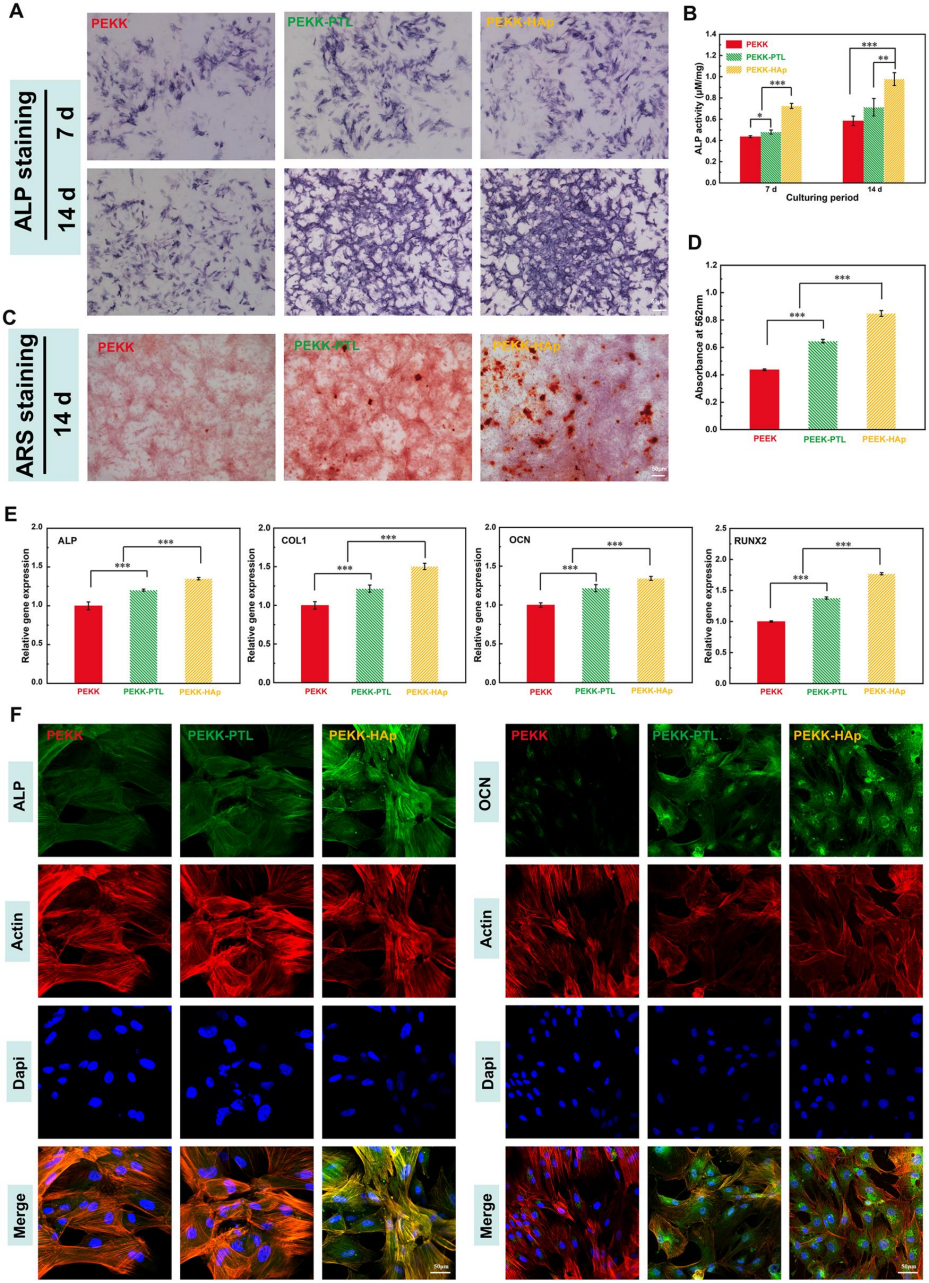
Osteogenic differentiation of rBMSCs cultured in different groups. (**A**) ALP staining of rBMSCs cultured with extracts from different scaffolds for 7 and 14 days. (**B**) Quantitative ALP activity determination. (**C**) Alizarin red staining of rBMSCs cultured with extracts from different scaffolds for 14 days. (**D**) Quantitative analysis of mineralized nodules. (**E**) Osteogenesis-related genes expressions of rBMSCs cultured for 14 days. (**F**) ALP and OCN immunofluorescent staining of rBMSCs after 14 days (^*^P < 0.05, ^**^P < 0.01 and ^***^P < 0.001).

Figure 6A illustrated a continuous increase in ALP staining intensity over time in all groups, with significantly greater staining observed in the PEKK-HAp group compared to the other two groups, consistent with the quantitative ALP activity (Figure 6B). Mineralization ability was observed by ARS staining, as shown in Figure 6C. After 14 days of culture, PEKK-HAp exhibited significant calcium deposition compared to the other groups, which was further supported by quantitative analysis conducted among the groups (Figure 6D).

In addition, the findings of PCR revealed a significant upregulation in the expression of osteogenesis-related genes, including COL1, RUNX2, ALP and OCN, in the PEKK-HAp group when compared to both the PEKK-PTL group and the pure PEKK group (Figure 6E). Finally, representative immunofluorescence staining images of bone-specific proteins were displayed in Figure 6F. Obviously, much stronger ALP and OCN marker signals were detected in the PEKK-HAp group than in the other groups, which appeared to support the results from the data analysis described above. The findings indicated that PEKK-HAp held promise as a potential alternative medical implant, as it possessed the capability to enhance the osteogenic differentiation of rBMSCs.

### *In vivo* osteointegration

**Figure 7.**
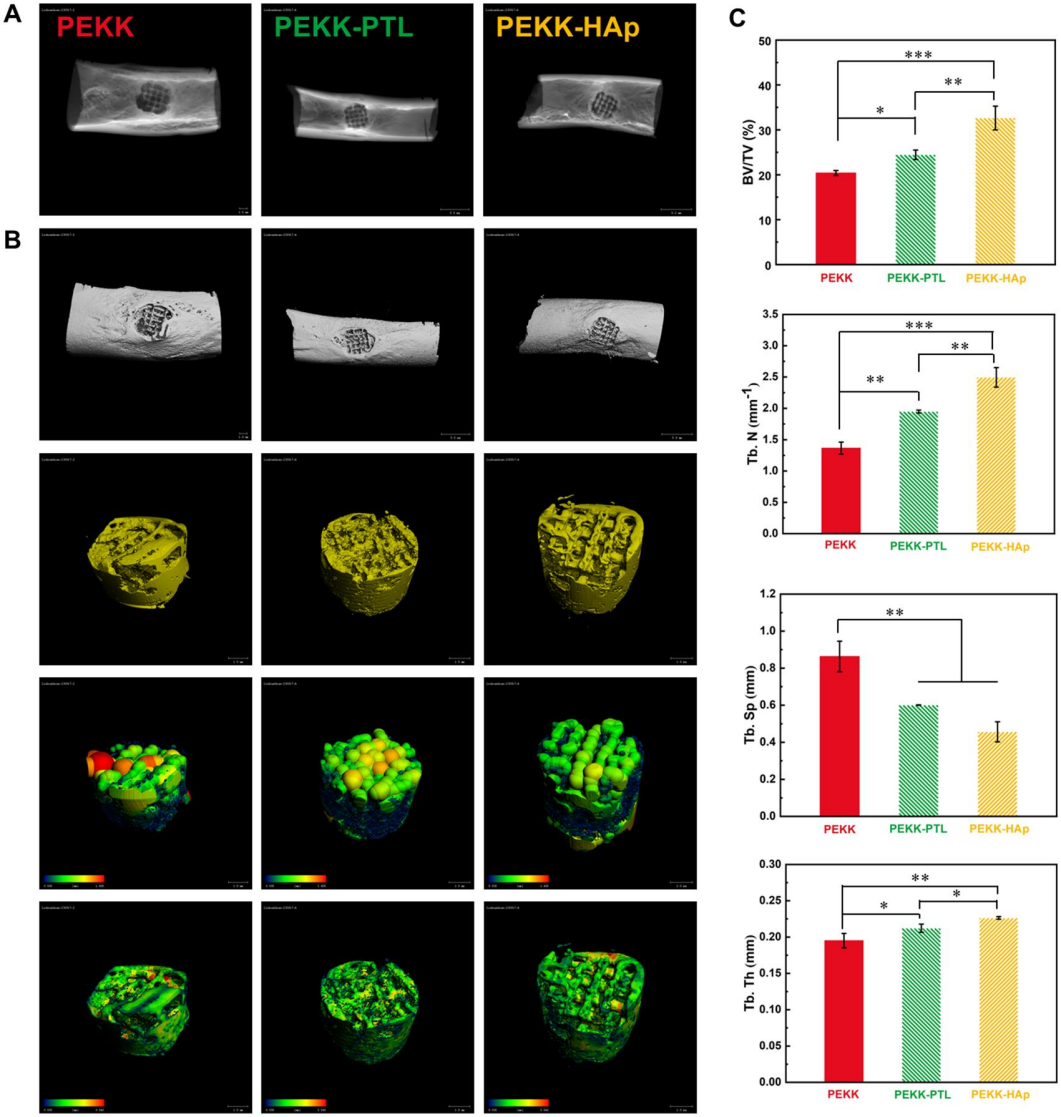
*In vivo* osseointegration study around scaffolds in femoral defect of a rabbit model. (**A**) X-ray images and (**B**) micro-CT reconstruction of newborn bone at 8-week postoperatively, respectively. (**C**) Quantitative analysis of micro-CT data, including BV/TV, Tb. Th, Tb. N and Tb. Sp (^*^P < 0.05, ^**^P < 0.01 and ^***^P < 0.001).

To assess the effect of the biomaterials on *in vivo* bone healing, a femoral defect model was established in rabbits, with the implanted scaffolds effectively filling the bone defect (Supplementary Figure S5). Micro-CT analysis was used to assess the growth of newly formed bone into the scaffolds after 8-week implantation. From X-ray images (Figure 7A) and 3D reconstruction (Figure 7B), scaffolds were transparent owing to radiolucency, the bone regeneration rate of the PEKK-HAp scaffold was faster than that of the other scaffolds, and more inner pore spaces were occupied by bone ingrowth in the PEKK-HAp scaffold. The bone ingrowth was assessed through quantitative analysis of the 3D reconstructed new bone tissue, including BV/TV, Tb. N, Tb. Sp, and Tb. Th (Figure 7C). It was evident that osseous tissues developed within PEKK-HAp scaffolds exhibited higher values of BV/TV, Tb. N, and Tb. Th compared to the other two groups, with the trend of these parameters as follows: PEKK < PEKK-PTL < PEKK-HAp. Additionally, PEKK demonstrated the highest Tb. Sp, representing the distance between adjacent trabeculae, among all experimental groups, indicating suboptimal ossification.

**Figure 8.**
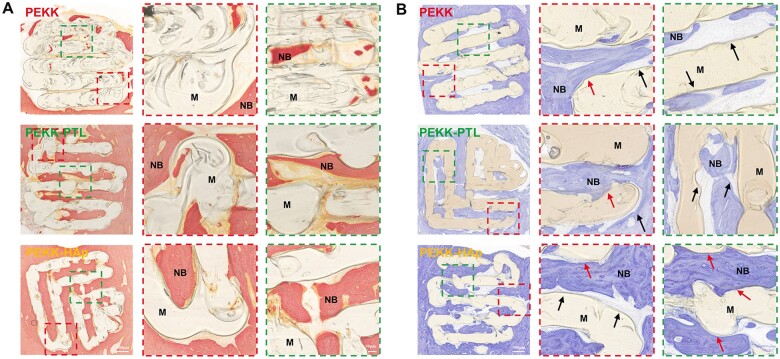
Histological assessment of femoral defects in a rabbit model. (**A**) Van-Gieson staining and (**B**) toluidine blue staining of the femoral defect samples in the PEKK, PEKK-PTL and PEKK-HAp groups at 8-week postoperatively, respectively. (NB, new bone; M, materials; black arrows mark the fibrous capsule; red arrows mark the direct contact between new bone and the implants).

Histological analysis was conducted on hard tissue slices stained with VG and TB to assess the osteointegration of the scaffolds. In VG staining images, the presence of dark red areas surrounding and within the scaffolds indicated regenerated bone (Figure 8A). The depth and volume of bone ingrowth were superior in the PEKK-HAp scaffold than in the PEKK and PEKK-PTL scaffolds, indicating better osseointegration in the PEKK-HAp scaffold. The findings of TB staining were in agreement with VG staining, revealing a greater presence of bluish-violet stained bone tissue around the margin of the PEKK-HAp scaffold than that in the other two groups. Enhanced integration between scaffolds and host bone was evident in the magnified views of the PEKK-HAp group (Figure 8B). In contrast, a noticeable void was observed between pure PEKK scaffolds and the adjacent host bone, and the gap of the PEKK-PTL scaffolds was greater than that of PEKK-HAp, indicating that HAp coating could trigger osteogenesis and osteoinduction, aligning with the results of a previous *in vitro* study.

## Discussion

Large bone defects have still been a major challenge in orthopedic treatment. Even the gold standard treatment, autologous bone grafting, presents certain limitations, including donor site morbidity and autogenous bone deficiency [32, 33]. Currently available ceramics and metals are expected to overcome this problem for bone repair [34, 35], however, their inadequate ability to regulate both mechanical characteristics and osteoinductive potential limit their clinical application. PEKK, as a new generation of implant materials, has been increasingly investigated because of its similar mechanical properties to natural bone and clinical potential after chemical modifications and structural alterations [4, 36]. Multiple studies in the field of bone substitutes have revealed that porous scaffolds with controlled geometry, connected pores and reasonable porous structures are favorable for osteogenesis [10, 11]. Inspired by these findings, we fabricated 3D porous biomimetic PEKK scaffolds using FDM manufacturing technology. The 3D printing process enhanced precision in the design of the structure, encompassing pore size, porosity and pore interconnectivity, which were essential for facilitating cell growth, proliferation and migration. In addition, the 3D structure of the required scaffold was fabricated using architectural data from the defective tissue, enabling greater structural complexity, flexibility and customization to meet patient-specific needs [37, 38]. The porous structure endowed the PEKK scaffolds with well-matched mechanical properties, less stress shielding, and a sufficient supply of nutrients and oxygen [39, 40]. The results of the mechanical tests demonstrated that the porous PEKK scaffolds exhibited a sufficiently high mechanical strength and an elastic modulus comparable to that of native bone, meeting the mechanical requirements for the repair of load-bearing bone defects and avoid bone resorption led by stress shielding. Additionally, an increase in pore size corresponded to a decrease in the mechanical properties of the samples. In addition to the evaluation of the scaffolds’ mechanical properties, their biological activity was systematically explored through well-designed *in vitro* experiments, including cell proliferation, ALP activity, and mineralization capability of rBMSCs. The findings from this study indicated that PEKK scaffolds with a pore size of ∼600 μm were the optimal pore size for bone substitutes.

Although the porous structure exhibits some bone ingrowth, it is a chemically and biologically inert material that might not effectively promote excellent osteointegration [41, 42]. Various techniques have been developed to fabricate HAp coating on bone implants. Unfortunately, precise control over the microstructures of the HAp coating and the promotion of strong mineral attachment to the substrate surfaces remain significant challenges, greatly impeding the successful fabrication and advancement of advanced hybrid biomaterials. The PTL film was utilized as the intermediate adhesive for the interfacial bonding between the organic (PEKK polymer) and inorganic (HAp nanoparticles later) components. Subsequently, a PTL-assisted HAp coating was conducted using a biomimetic coating technique to enhance the osteointegration and osteogenesis of porous PEKK scaffolds. This simple and functionalized approach began with the reduction of the disulfide bonds in the lysozyme chain induced by TCEP under physiological conditions. Then, the α-helix structure of lysozyme unfolded into a β-sheet configuration, resulting in the formation of amyloid nanospheres. The PTL layer exhibited stable adherence to the surface of the PEKK scaffold through physical entanglement, hydrophobic interactions and hydrogen bonding [30]. The durability of the initial PTL layer on the PEKK surface would provide a robust foundation for subsequent fabrication of HAp coating. Following classical crystal growth theory, the PTL layer, with enriched functional groups, chelated positively charged Ca^2+^ via electrostatic interactions [43]. After a 3-day incubation in SBF, lath-like clustered protrusions of HAp crystals developed at nucleation sites. This process represented a straightforward, effective, cost-efficient and environmentally friendly approach to substrate surface functionalization compared to other traditional surface pretreatment techniques. The surface topography, elemental composition and chemical groups of the printed scaffolds were systematically characterized by AFM, FITR, XRD, XPS and SEM-EDS. The results exhibited noticeable alterations in the surface morphology and the characteristic peak, indicating that HAp coating was performed and uniformly distributed on the PTL surface with strong interactions between PTL and HAp during the biomimetic mineralization process. The resulting HAp coating markedly enhanced the roughness and hydrophilicity of PEKK-HAp, which may exert a favorable influence on cellular responses and bone growth. Yuan et al. [1] fabricated a rough surface with nanotextures by the porogen leaching method and sulfonation treatment, observing improved cell adhesion and osteointegration properties in the modified biomaterials. Our experimental findings demonstrated that the PEKK-HAp group scaffolds facilitated the proliferation and adhesion of rBMSCs cells, supporting the bioactive characteristics of HAp documented in existing literature [28]. Subsequently, the effects of the scaffolds on cell migration were investigated through scratch assay and transwell assays, revealing that the porous PEKK scaffolds modified with a HAp coating enhanced the migration ability of rBMSCs compared to unmodified and PTL-modified PEKK scaffolds. The interaction between cells and material surfaces profoundly influenced implant biocompatibility, with cell response being crucial for achieving an optimal host-implant reaction [21]. Thus, the conclusions were made that the micro/nanostructured surface of the PEKK-HAp scaffolds had a positive influence on cellular behavior, thereby promoting *in vivo* osteointegration. Furthermore, the addition of HAp stimulated the expression of relevant osteogenic genes (ALP, OCN, RUNX2, COL-1) and proteins (ALP, OCN) detected by quantitative RT–PCR and immunofluorescence staining and stimulated the mineralization of rBMSCs, contributing to the enhancement of the osteoinductive capability. A rabbit femur defect model was subsequently developed to investigate osteoinduction and osteointegration ability of these scaffolds *in vivo*. The growth and distribution of new bone tissue inside scaffolds and the surrounding tissue were systematically investigated by micro-CT and histological staining. The micro-CT results confirmed that the bone volume fraction within the scaffolds was greater in the PEKK-HAp and PEKK-PTL groups than in the PEKK group after implantation in the femoral bone for 8 weeks, demonstrating that modifying the physical structure and surface chemistry of bioinert PEKK could impart exceptional osteoinductive properties. Histologically, the evaluation of tissue ingrowth and integration of scaffolds was performed using TB and VG staining techniques applied to hard tissue sections. These findings provided evidence that the porous structure and strong osteoinductive properties of the PEKK-HAp scaffold could accelerate osteointegration with host bone and promote the growth of bone tissue.

The enhanced bone formation induced by PEKK-HAp could be attributed to the combined stimulating effects of its porous architecture and surface composition resembling bone apatite. The porous structure increased the scaffold’s specific surface area, facilitating the transport of oxygen and nutrients, which in turn promoted the recruitment, adhesion and proliferation of rBMSCs [39, 44]. Additionally, the surface roughness and the release of Ca^2+^ ions from the stable apatite layer on the surface effectively promoted the differentiation of BMSCs and bone mineralization [45, 46]. Many studies confirmed that incorporating bioactive HAp into materials could stimulate the secretion of bone-related cytokines in BMSCs to promote osteogenic differentiation, resulting in bone formation [47, 48].

Notwithstanding our endeavors, the present study possesses limitations and needs to be further investigated. First, as only scaffolds with uniform pore structures have been evaluated for osteogenesis, future investigations should focus on fabricating and evaluating scaffolds with diverse cellular architectures, especially a well-organized gradient structure mimicking genuine bone tissues [44, 49]. Additionally, considering the translational potential of 3D-printed scaffolds for clinical applications, the long-term *in vivo* stability and biological safety of the scaffolds are crucial and need to be clarified [50].

In summary, we herein demonstrated that macroporous architecture and bone-like apatite coating are promising methods to alter the biological properties of PEKK implants from bioinert to osteoinductive. The biomimetic coating process utilized in this study was characterized by its simplicity and efficiency, resulting in a bioactive porous PEKK-HAp scaffold, which was confirmed as a promising candidate for repairing load-bearing bone defects.

## Conclusion

In this study, five types of porous PEKK scaffolds with varying pore sizes were successfully designed and manufactured using the advanced 3D printing technique. Evaluation of their mechanical properties and examination of *in vitro* outcomes indicated a correlation between pore size and the proliferation as well as differentiation of rBMSCs on the scaffold surfaces. Porous PEKK scaffolds with a pore size of ∼600 μm showed balancing mechanical functionality with biological performance of cell adhesion and osteogenic differentiation. Subsequently, the surface of the PEKK scaffold was modified by PTL, which acted as an effective template to lead to the formation of functional structural surfaces. Indeed, the surface roughness and hydrophilicity were significantly improved after modification. Additionally, bioactive HAp film enhanced adhesion, proliferation and osteogenic differentiation. Furthermore, when implanted *in vivo* in a rabbit femoral cavity defect model, PEKK-HAp demonstrated superior bone formation and osseointegration compared to PEKK. Collectively, these findings established a solid foundation for the design and fabrication of porous PEKK-based scaffolds with appropriate geometries to facilitate osteogenesis. Moreover, combining a biomimetic mineralization strategy with 3D printing technology, customized and bioactive PEKK-HAp scaffolds were developed, showing great potential as candidate for bone defect. Overall, this new synergetic strategy makes significant advancements in resolving the osteoinductive puzzle of synthetic biomaterials, which can contribute to the development of a new generation of polymeric biomaterials and devices with enhanced osteoinductive properties.

## Supplementary Material

rbae023_Supplementary_Data
