# Supplementary material for: Bio-functional hydroxyapatite-coated 3D porous polyetherketoneketone scaffold for enhanced osteogenesis and osteointegration in orthopedic applications
Source: Regen Biomater. 2024 Mar 14;11:rbae023. doi: 10.1093/rb/rbae023 (PMC10980557; doi:10.1093/rb/rbae023)
Supplement: rbae023_Supplementary_Data [file rbae023_supplementary_data.docx]

**Bio-functional Hydroxyapatite Coated 3D Porous Polyetherketoneketone Scaffold for Enhanced Osteogenesis and Osteointegration in Orthopedic Applications**

**
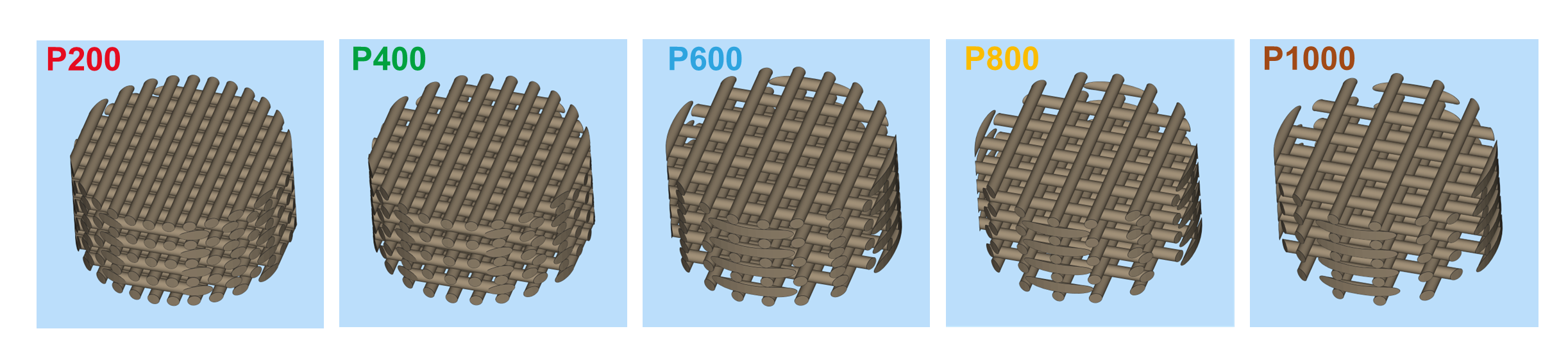
**

**Figure S1.** Porous PEKK scaffolds with different pore sizes and porosities in the CAD model.


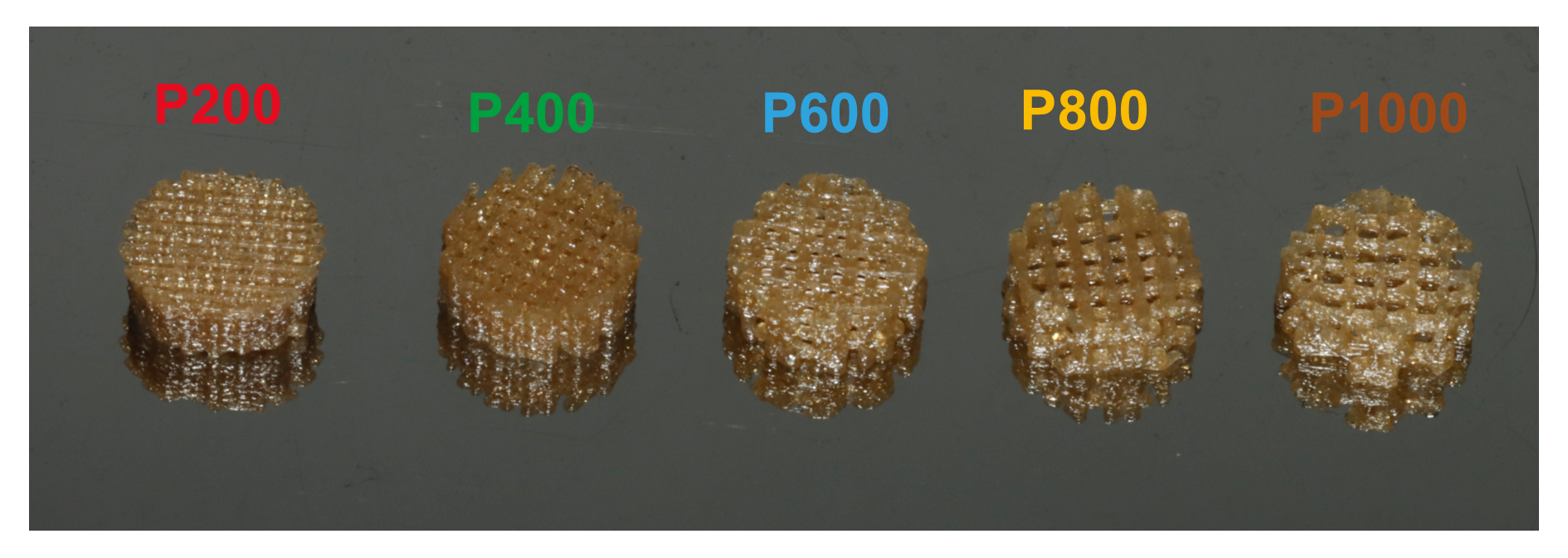
**Figure S2.** Gross images of 3D printed porous PEKK scaffolds with a size of Φ 10 mm × 3 mm.


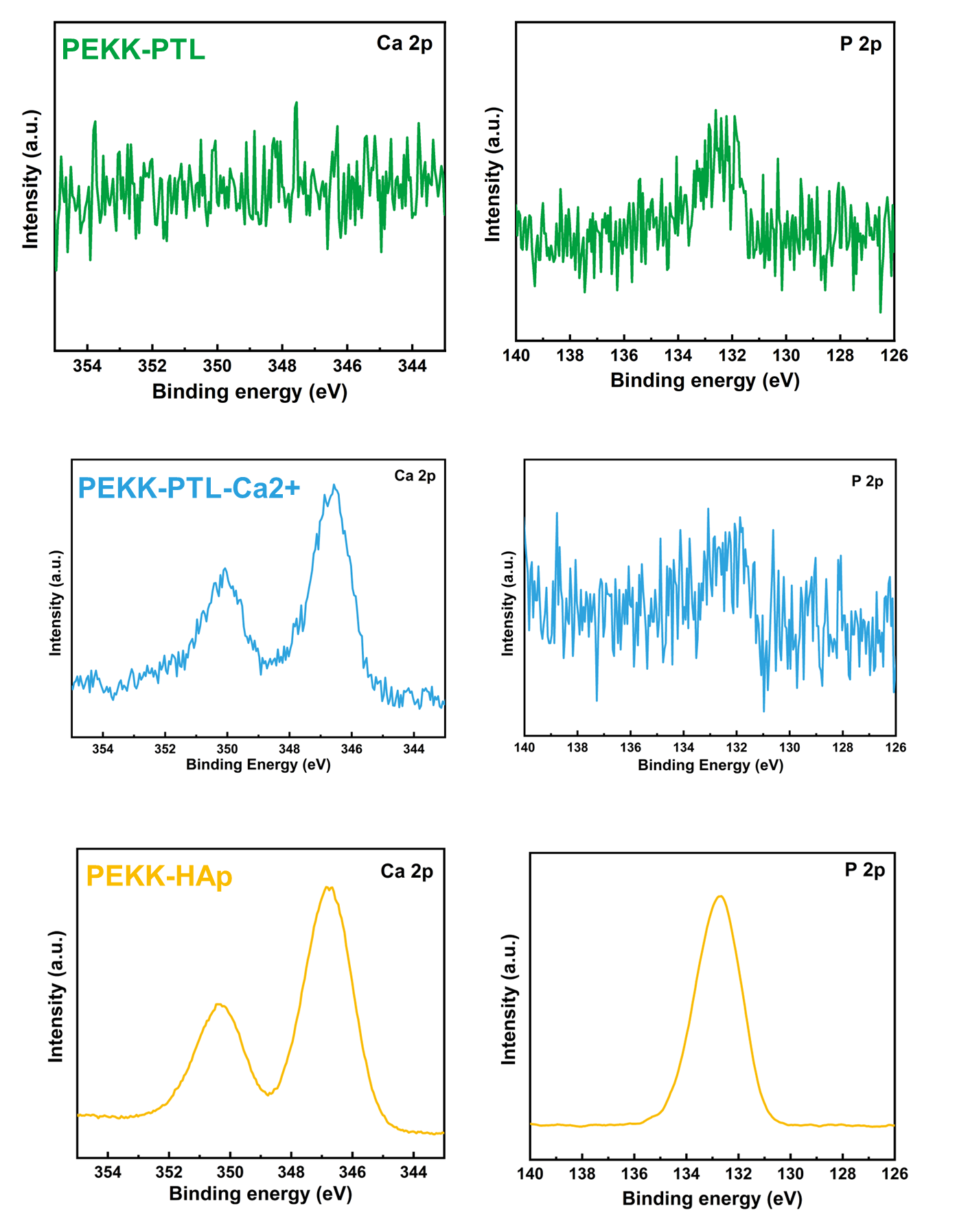


**Figure S3.** High-resolution spectrum analysis of Ca 2p and P 2p peaks on PEKK-PTL, PEKK-PTL-Ca^2+^ and PEKK-HAp scaffolds.


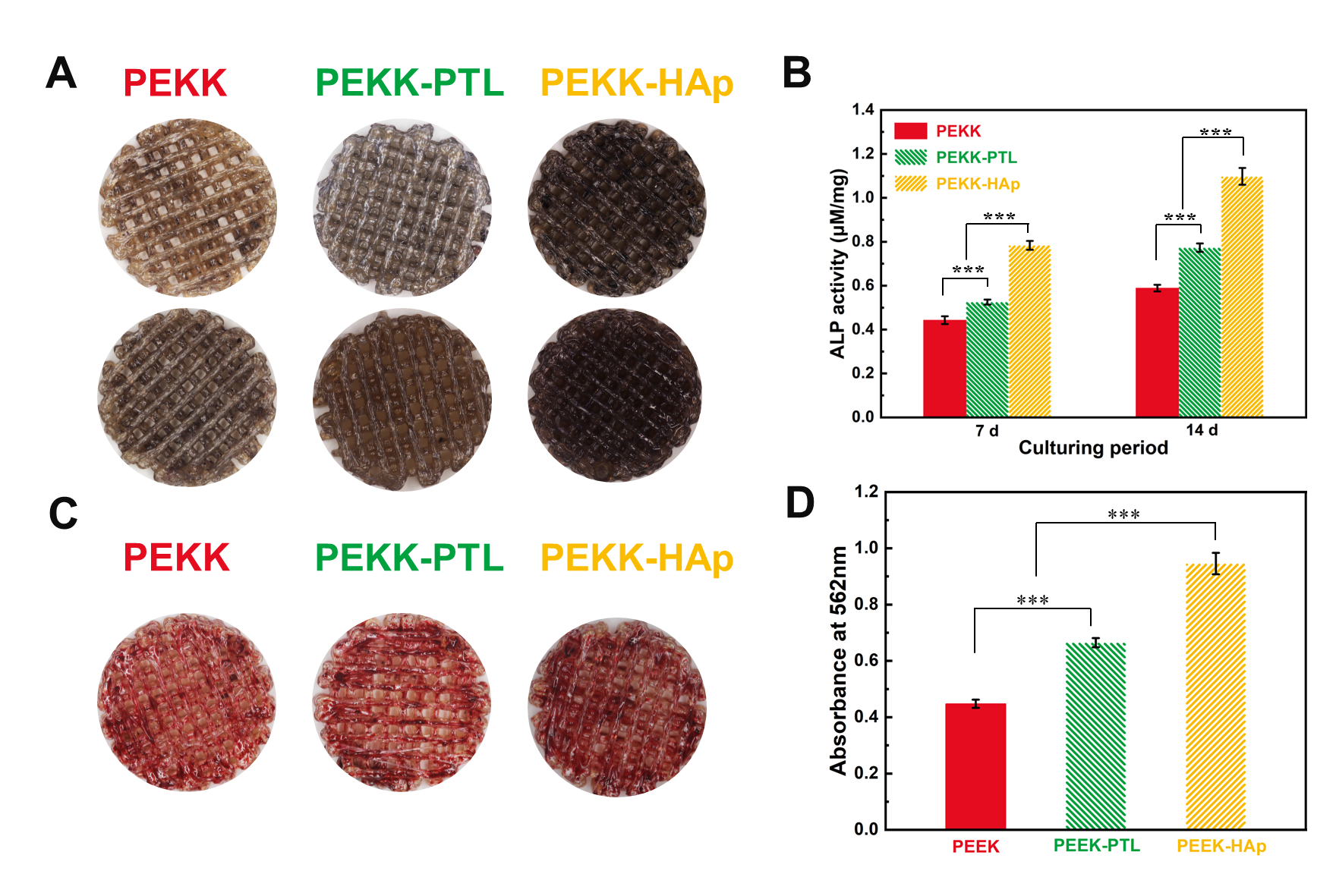


**Figure S4.** (A) ALP staining and (B) quantitative activity of rBMSCs cultured on the different scaffolds for 7 and 14 days. (C) ARS staining and (D) absorbance values of calcium deposition of rBMSCs cultured on the different scaffolds for 14 days.

**
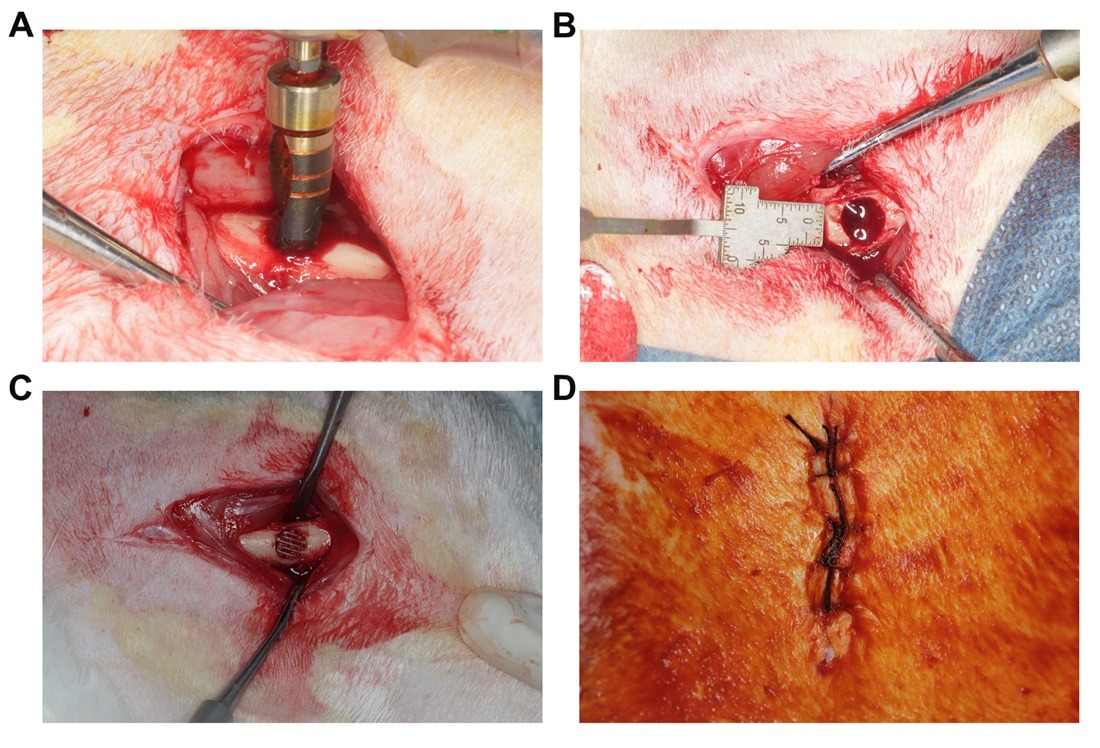
**

**Figure S5.** Schematic diagram of femoral defect and snapshots of the surgical procedure.


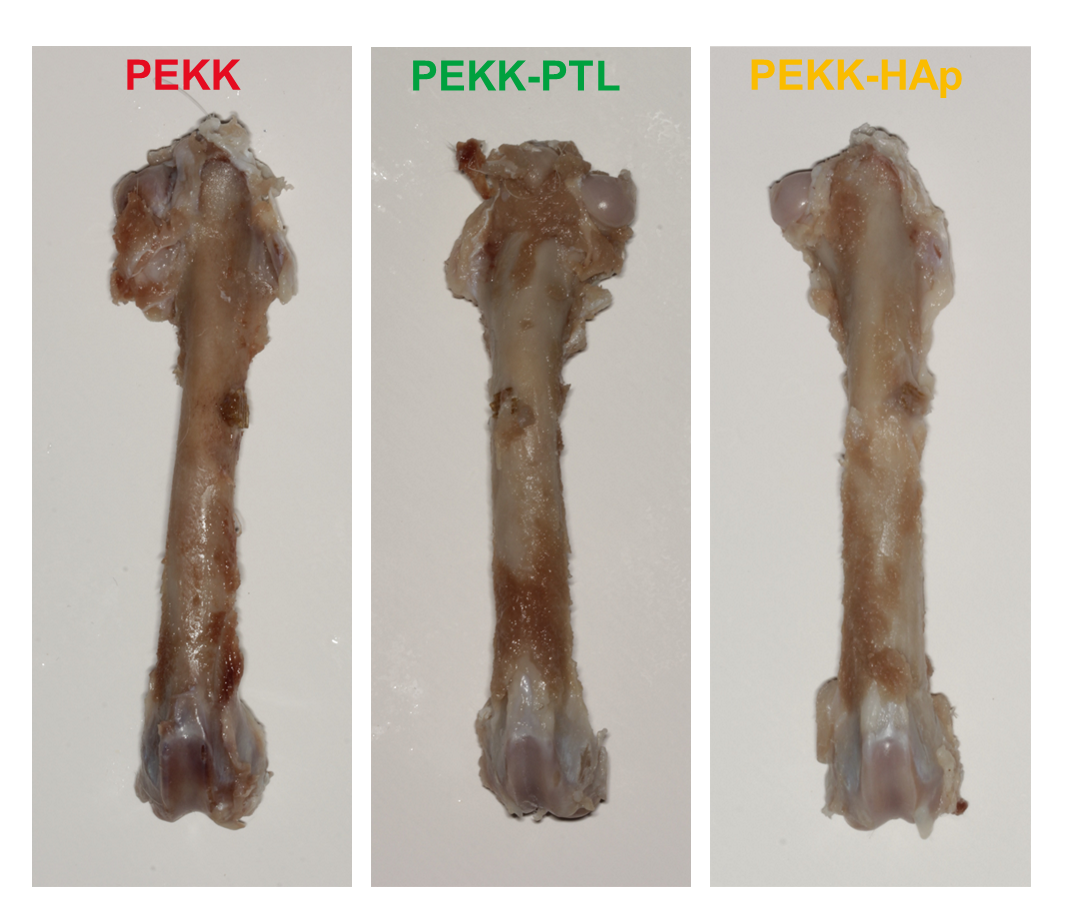


**Figure S6.** Representative images of femurs from rabbits in different groups 8 weeks after surgery.

**Table S1.** Primers used in the RT-PCR of rBMSC cells.

| **Genes** | **Primer sequences** |
| --- | --- |
| ALP | Forward: 5′-ATGCTCAGGACAGGATCAAA-3′  Reverse: 5′-CGGGACATAAGCGAGTTTCT-3′ |
| COL1 | Forward: 5′-AGCTCGATACACAATGGCCT-3′  Reverse: 5′-CCTATGACTTCTGCGTCTGG-3′ |
| OCN | Forward: 5′-CAGACAAGTCCCACACAGCA-3′  Reverse: 5′-CCAGCAGAGTGAGCAGAGAG-3′ |
| RUNX2 | Forward: 5′-ATCATTCAGTGACACCACCA-3′  Reverse: 5′-GTAGGGGCTAAAGGCAAAAG-3′ |
| β-actin | Forward: 5′-CCTCTATGACAACACAGT-3′  Reverse: 5′-AGCCACCAATCCACACAG-3′ |

**Table S2.** Characterization of porous PEKK scaffolds with different pore sizes.

| Parameters | Groups | | | | |
| --- | --- | --- | --- | --- | --- |
|  | P200 | P400 | P600 | P800 | P1000 |
| Actual pore size (μm) | 225±9.8 | 411±22.1 | 596±23.4 | 786±24.2 | 993±26.0 |
| Actual porosity (%) | 24.6 | 33.7 | 40.9 | 48.0 | 58.6 |
| Interconnectivity (%) | 100 | 100 | 100 | 100 | 100 |
